# Evaluation of free-floating bike-share on a university campus using a multi-method approach

**DOI:** 10.1016/j.pmedr.2019.100981

**Published:** 2019-08-28

**Authors:** Debra Kellstedt, John O. Spengler, Katie Bradley, Jason E. Maddock

**Affiliations:** Texas A&M University School of Public Health, United States of America

**Keywords:** Bicycling, Transportation, Built environment

## Abstract

Bike-sharing, especially free-floating bike-share, has tremendous potential for increasing active transport on a college campus. Increased bike use improves public health, reduces pollution, and solves traffic congestion problems. Like other innovations, free-floating bikeshare proceeds through various stages while disseminated and before being widely adopted and accepted. A multi-method study using quantitative bike usage data, a cross-sectional survey, and focus group discussions was used to evaluate the Spring 2018 launch of a free-floating bike-share program at a large public university. Three months after implementation, there were 19,504 registered users, 24,371 different riders, 165,854 rides, and 85,778 miles traveled. The average trip length was 0.52 miles and lasted 8.3 min. Survey data from 2845 students, faculty, and staff revealed that 33.6% had used the bikes. Bike users were more likely to be students, freshmen, living on campus, be a current biker, and have confidence in their biking ability. Focus groups revealed that safety was a concern, knowledge about how the program worked was low among non-users and faculty and staff, cost was a barrier, and that adherence to bike-share rules needed to be improved. A large segment of the university population quickly adopted free-floating bike-share. However, continued work needs to be done to enhance safety, provide clear guidelines on bike-share rules (e.g., bike parking), and increase knowledge of the program with a specific focus on use by faculty and staff to ensure continued success and ultimately improve health.

## Highlights

• Almost 1/3 of a university population quickly adopted free-floating bike-share.
• Just after launch, there were 19,504 registered users and 165,854 rides.
• Users were more likely to be students, freshmen, males, and those living on campus.
• Concerns remained about safety and adherence to rules of the program.

## 1 Introduction

Bicycling as active transport provides important health benefits, reduces pollution, and alleviates traffic problems (de Nazelle et al., 2011; Pucher et al., 2010; Weaver and Garber, 2011). Increasing physical activity (PA) levels through cycling has potential to improve heart health, lower BMI, and reduce type 2 diabetes (Oja et al., 1998; Wannamethee et al., 2000). Cycling also has the dual benefit of improving health and providing efficient transportation options (Lewis et al., 1997).

The U.S., Canada, and several European countries have seen growth in bike use over the past twenty years (Pucher and Buehler, 2011; Su et al., 2010; Xing et al., 2010), and the acceptance of cycling for transport, while still low, has increased over time as well (Zhang et al., 2016). According to a 2012 national survey, 7% of those who bike do so for commuting to and from work, and 4% for commuting to and from school (Schroeder and Wilbur, 2012).

While some may use personal bikes for transportation purposes, new bike programs have been developed to allow individuals to share and use bikes they do not personally own. The concept of bike-sharing started in the 1960s, but problems with theft led to slow growth until tracking technology improved in the 1990s (DeMaio, 2009). Station-based, or docked, bike systems were developed to allow fleets of bikes to be checked out (with coins, credit cards, and now mobile phones), used for a period of time, and then returned to docking stations (Fishman, 2016). By 2009, there were over 120 station-based bike-share systems around the world (DeMaio, 2009; DeMaio, 2018), and as of 2018, there was station-based bike-share in most major cities in the U.S. (Hirsch et al., 2019a). In 2015, a new technology using free-floating, or dockless bikes, became available (Tian et al., 2018). Free-floating bike-share was brought to the United States in 2017 with three free-floating bike-share companies launching in Seattle, Washington (Hirsch et al., 2019a, Hirsch et al., 2019b).

Free-floating bike-share relies on global positioning systems (GPS) to track bikes, and mobile phones for sign-up and payment. Some bikes use a mobile-controlled wheel lock, so bikes can be left in any designated area, without needing to be station-based. Other strengths of free-floating bike-share include the ease of short-term use at a relatively low cost (Shaheen et al., 2014). Expensive docking stations are not necessary in this innovation, driving down the cost to $1–2 per ride (Mooney et al., 2019). These programs are able to offer more bikes than are available with station-based programs, providing communities increased access to bikes (Hirsch et al., 2019a, Hirsch et al., 2019b). While more bikes may be available, a challenge is the distribution of the bikes. Not all communities may access them if the bikes are only left in certain areas (Hirsch et al., 2019a, Hirsch et al., 2019b; Mooney et al., 2019). To address this issue, free-floating bike-share companies employ redistribution efforts to move bikes to places they may be needed. Other challenges of the innovation are the bikes being left in undesirable places, like blocking sidewalks, and safety problems, like no provision of helmets (Hirsch et al., 2019a, Hirsch et al., 2019b).

Large universities face challenges moving people across campus efficiently. Studies show benefits of cycling on college campuses in helping to alleviate parking and transportation problems (Arnott and Inci, 2006; Balsas, 2003; Shang et al., 2007). In 2015, over 50% of college students had ridden a bike in the past year (American College Health Association, 2018). As of 2009, >18 million young adults in the U.S. attended some form of college (Snyder, 2010). This population may be accepting of cycling, especially if bikes are accessible. Other studies have confirmed that cycling, among adults is most popular among the youngest age category (16–24 year olds), males, those who use public transit, and those who are physically active (Harris, 2011; Moudon et al., 2005). According to the Rogers Diffusion of Innovation Theory, younger populations are also more likely to adopt new technologies (Rogers, 2003).

Given the introduction of the newest bike-share technology, college campuses have a unique opportunity to address a large number of young adults at a unique point in time, in a defined space, to promote cycling behaviors. Implementing a free-floating bike sharing system may promote PA through increased bike usage not only among students, but among faculty and staff as well. This is the first study to our knowledge evaluating the use of free-floating bike-share on a college campus. This study examined usage and factors predicting bike-share adoption. We also explored themes related to bike-share use on campus after the free-floating bike-share system launch.

## 2 Methods

### 2.1 Setting

This study took place at a large land-grant university that covers over eight square miles of university-owned land. The university has over 68,000 students and 3039 faculty and 7306 staff (Texas A&M University, 2018).

### 2.2 Study design

In Spring of 2018, a large public university piloted a free-floating bike-share program through a public-private partnership with a bike-share company. The program, one of the largest on a college campus, supplied 850 bikes with an additional 2150 projected for the following Fall semester (Steedly, 2019). This number of bikes calculated to one bike per 92 people during the launch and one bike per 26 people once the full fleet was on campus. A multi-method study was used to assess bike usage, non-usage, user characteristics, and unintended consequences. Three types of data were collected: bike usage, a quantitative survey, and focus groups of users and non-users of the bikes. Study protocols were approved by the University Insitutional Review Board, and focus group participants provided consent with a signed information sheet. Survey participants provided informed consent by reading about the study and clicking on the survey link provided.

### 2.3 Bike usage data

The bike-share company collects ongoing usage data about bike ridership based on GPS units on the bikes and participant app sign-ups. Data were used to calculate registered users, number of rides, and total miles traveled between launch on February 27, 2018 through the end of May 2018.

### 2.4 Quantitative surveys

In April 2018 an online Qualtrics survey link was emailed to over 75,000 university students, faculty, and staff, and of those, 26,267 emails were opened. Participants were eligible if they were over 18 and either student, faculty, or staff at the university. Within a two-week period, 3219 surveys were returned for analyses, with a response rate of 12.3%. The brief 10-item survey asked about class rank, campus residency, employment status, age, gender, current bike usage, cycling confidence, and bike-share usage.

#### 2.4.1 Survey measures

The following survey measures are detailed in the supplemental materials: bike-share use, campus residency, employment status, class rank, age, gender, current biking, and biking self-efficacy.

#### 2.4.2 Analysis

Of the 3219 surveys returned, 2845 responded to the bike use question and were retained for further analysis. Chi-square tests were conducted to examine associations between respondent type and bike-share usage versus non-usage. Logistic regression analyses were conducted on student data only since few faculty and staff had used the bikes. A dichotomous outcome variable of bike-share usage was used to determine associations with residency on campus, class rank, gender, current bike usage, and cycling confidence. Table 1 shows categories within each variable. Both current bike usage and cycling confidence were collapsed into yes/no categories. Results of logistic regression analyses are presented as odds ratios and 95% confidence intervals (CI). Missing data occurred in <2% of cases that completed the bike use question. Therefore, we used only complete cases for the analysis and did not use methods to estimate missing values. All analyses were performed in Stata/SE 15.1.

**Table 1:** Bike-share user demographics (n = 2845).

Variable	% who have used bike-share (n)	% who have not used bike-share (n)
Campus residency [χ^2^ (1) = 65.5, p < .001]		
Student off campus	37.0 (554)	63.0 (944)
Student on campus	56.7 (322)	43.3 (246)
Employment status [χ^2^ (1) = 3.3, p = .071]		
Staff	9.1 (54)	90.9 (542)
Faculty	13.7 (25)	86.3 (158)
Class rank [χ^2^ (4) = 76.9, p < .001]		
Freshman	55.9 (241)	44.1 (190)
Sophomore	47.8 (170)	52.3 (186)
Junior	41.7 (194)	58.3 (271)
Senior	27.4 (116)	72.6 (308)
Graduate student	39.7 (155)	60.3 (235)
Gender [χ^2^ (2) = 29.3, p < .001]		
Female	29.5 (488)	70.5 (1164)
Male	39.2 (461)	60.8 (714)
Current biking [χ^2^ (1) = 448.7, p < .001]		
Yes currently biking	55.7 (667)	44.3 (531)
No not currently biking	17.6 (288)	82.4 (1348)
Confidence in biking ability [χ^2^ (1) = 113.6, p < .001]		
Yes confident	38.6 (858)	61.4 (1365)
No not confident	15.7 (97)	84.3 (521)

### 2.5 Focus groups

#### 2.5.1 Recruitment

Survey respondents (n = 466) indicated willingness to participate in an hour-long focus group. Participants were recruited from this list via email in order of responses until sessions filled to capacity. Thirty-five survey respondents participated in the focus groups, 46% of whom were female. In May 2018, four focus groups were held: one for students who had tried the bike-share (n = 10), one for students that had not tried the bike-share (n = 8), one for faculty and staff who had tried bike-share (n = 8), and the other for staff/faculty who had not tried (n = 9). The number of focus groups was based on researchers' and participants' end-of-semester availability and scheduling prior to the university exam period.

#### 2.5.2 Data collection

Focus group participants were asked about their perceptions of and experiences with bike-share and cycling around campus. Two semi-structured focus group discussion guides asked about experiences with bike-share and biking, one for bike-share users on campus and another for non-users of campus bike-share. These guides are shared in the supplemental materials.

Two researchers trained in qualitative research methods conducted the focus groups. Focus groups were audio-recorded, and consent was obtained prior to the start of the discussions with a signed information sheet.

#### 2.5.3 Analysis

Recordings were transcribed verbatim and coded using thematic analysis (Burnard et al., 2008). An initial coding scheme was developed by two interviewers based on one separate transcript for each. The coding scheme was updated after additional transcripts were coded. Interviewers coded each transcript independently. After an iterative coding process and several meetings, consensus was reached on emerging themes and corresponding quotes. Results are presented as themes and quotes in Table 3. Coding was done using qualitative software NVivo 12.0.

## 3 Results

### 3.1 Overall bike usage

Over three months, there were 19,504 registered users, 165,854 rides, and 85,778 miles traveled. The average trip length was 0.52 miles and lasted 8.3 min.

### 3.2 Participants

The sample consisted of 2845 survey respondents. Seventy-three percent (n = 2066) of respondents were students. Six percent of total respondents identified as faculty (n = 183), and 21% indicated they were staff (n = 596). Seventy-three percent of students responded currently living off campus (n = 1498) and 28% responded living on campus (n = 568). Fifty-eight percent of respondents were female (n = 1652).

### 3.3 Descriptive and bivariate analyses

Table 1 highlights bike-share use by respondent characteristics. Nine hundred and fifty-five respondents used the bike-share (33.6%). The overall student usage rate was 42.4% (n = 2066). Of those students living on campus who responded to the survey, 56.7% reported that they used the bikes (n = 322) as compared to 37% of student survey respondents who used the bikes and lived off campus (n = 554). Staff were less likely than faculty to have used bike-share. Among students, seniors (27.4%) used bike-share the least, with almost twice as many freshmen (55.9%) using bike-share. Overall, females reported using the bikes less often than males (29.5%, vs. 39.2%, p < .05). Additionally, bike-share usage was significantly more popular with current bike riders, as opposed to those that did not regularly ride a bike (55.7%, vs. 17.6%, p < .05). Further, those who felt confident that they could safely ride on campus were more likely to have used bike-share than those who were not confident (38.6% vs 15.7%, p < .05).

### 3.4 Logistic regression

As shown in Table 2, statistically significant factors (p < .05) included campus residency, student class rank of senior or graduate student, current biking, and confidence in biking.

**Table 2:** Predictors of bike-share use.

Used bike-share	OR	95% CI OR	p
Campus residency-off	0.61	0.47, 0.79	<.001
Female	0.94	0.77, 1.15	.54
Class rank [ref: freshman]			
Sophomore	1.0	0.72, 1.42	.95
Junior	0.79	0.57, 1.11	.17
Senior	0.44	0.31, 0.63	<.001
Graduate	0.69	0.48, 0.99	<.05
Current biking-yes	3.1	2.49, 3.77	<.001
Confidence in biking-yes	2.2	1.65, 2.89	<.001

Note. OR, odds ratio; CI, confidence intervals.

### 3.5 Focus group results

Results indicated varied opinions about bike-share on campus. Bike-share users tended to be more positive than those who had not tried bike-share. The following themes emerged from the four groups: 1) bike safety; 2) bike-share program knowledge; 3) unintended consequences; and 4) cost and savings. The themes and accompanying quotes are summarized in Table 3.

**Table 3:** Themes identified by staff, faculty, and students: bike-share use and biking (n = 35).

Theme	Subtheme	Example quotes
Bike safety	Infrastructure	“One problem is that the streets here are designed in a way that seems to be intentionally insulting to bicycles in many cases.” (Faculty bike-share rider) “We just need the infrastructure for a bike culture in this town. And I think we can do it. They're already making strategies towards that so some of it's just going to take time. But I think a lot of it's just going to be education and establishing the culture.” (Staff non-rider)
	“types of riders”	“…you hit the nail on the head when you said it increases the number of bicycles being used by people who aren't bicyclist per se.” (Staff non-rider) “…I don't see how either the company or the university can instill the level of personal responsibility that's required to make these things safe because there's no way.” (Student non-rider)
	Helmets	“I tend to shy away from them, because there's no helmets.” (Staff bike-share rider)
Bike-share program knowledge	About the Company	“[the bike-share] claims that it will decrease car usage. I don't see that. I see it decreasing foot traffic and increasing bicycle traffic.” (Faculty non-rider) I would not ride that because some random company just dropped off these bikes here and trying to—I don't know—exploit college students who don't have a lot of money or something like this. (Student non-rider)
	Rules	“I think the students are going to have to learn some responsibility with them and some rules. Or else we're going to get real tired of them.” (Staff bike-share rider)
Unintended consequences	For Students	“…these bikes are a menace and they're all over the city.” (Student non-rider)
	For Faculty	“I've had a lot of people be cautionary to me from other places, colleagues and friends, seeing that these end up where the bikes go to places where people aren't, don't need them, like to the neighborhoods and stuff and so then they get left there and then they're not where you need them.” (Staff bike-share rider) “our bike rack for our customers to use is full of [bike-share] bikes and so my staff and we can't use the racks that are close to us.” (Staff bike-share rider)
Cost and savings	Free vs. Fee	“…basically during that free time frame, I rode, I don't know, four or five times around, just to use them. Kind of get familiar with them. I will probably grab one to ride back across to where I need to be after this thing's over if there's one sitting out there.” (Staff bike-share rider) “All I really knew is that it was really popular at first because it was free. I guess it was on trial period. And I know it's not as sought out anymore because you have to pay for it now.” (Student bike-share rider)
	Time	“So it's like, if I walk, it at least takes me 10–15 min. So this bike is very convenient.” (Staff bike-share rider) “It's just really convenient for exercise too and not having to like wait 30 min for the bus.” (Student bike-share rider) “I use it pretty much every day. I think that even when the bus service ends. So sometimes I stay late in my office like till midnight so I use the bike to ride home. It usually takes 10 min for me to reach home from my office.” (Student bike-share rider)

#### 3.5.1 Bike safety and infrastructure

Concerns about the safety of bike-share were echoed throughout the groups. Participants discussed the lack of cycling infrastructure and described drivers' lack of awareness in sharing the road. Other concerns for safety centered around the “types of riders” who use the bike-share. Specifically, those drawn to bike-share may not be experienced at cycling and not know how to ride safely. Insinuations were that these types of riders were not current bike owners/riders. Additional concern was voiced about riders not being responsible with the bikes, since they did not own them. Current bike riders were negative about sharing riding space with bike-share riders. Helmet use and how bike-share does not easily support this safety feature was discussed.

#### 3.5.2 Bike-share program knowledge

Confusion was expressed about the program. Some had no idea the program was university-sanctioned, and others lacked information about how the bike-share worked. Some participants were suspicious of the bike-share company, because it w as not an American company. In contrast, some participants (mostly bike-share users) described how they took time to research the program and investigated the app fully. Some talked about how rules do not matter if the consequences for breaking them are minor.

#### 3.5.3 Unintended consequences

Participants described how bike rack space was being used by bike-share and left little room for regular bikes. Some expressed concerns that free-floating bikes were “everywhere,” being left outside of appropriate parameters. Participants discussed pranks where students (presumably) left bikes in inappropriate places, like in trees on campus. Finally, concerns included the clutter of the bikes and how bikes were not being parked in proper places.

#### 3.5.4 Cost and savings

Students talked about program cost. Some used the bike-share when it was free, but used it less when they had to pay. Students described how the bus system on campus is free, so paying for something else was not desirable. Conversely, bike-share users liked the convenience and time-saving features. Drivers to campus liked that they could find a bike near the parking lot and bike to their building, instead of walking.

## 4 Discussion

After examining usage and factors predicting bike-share adoption and exploring themes related to bike-share use, findings suggest that a substantial number of people in a campus community used the bike-share. Within three months of piloting the program and according to the bike-share company data, about a third of the campus population were registered users (n = 19,504) with over 165,000 rides. In addition, almost 33.6% of survey respondents reported that they had used the bike-share program. This finding supports national data on the popularity of bike-share programs. A 2017 report notes the increasing uptake of bike-share around the U.S. with 34 million trips (National Association of City Transportation Officials, 2018). Similar to this study's findings, studies of the first free-floating bike-share launch in the U.S. in Seattle found that about one third of the adult population with internet access had used the bikes at least once during the six-month pilot period (Hirsch et al., 2019a, Hirsch et al., 2019b; Mooney et al., 2019).

Our survey results showed that freshmen and those living on campus were significantly more likely to use the bike-share program. These two groups are not mutually exclusive. At this university, typically only freshmen and a small amount of upperclassmen live in housing located on the main campus. The fact that freshmen may live on campus and that they are the youngest age group on campus could be contributing factors in their acceptance of a new innovation. The DOI theory posits that younger populations will be the innovators and early adopters of new innovations (Rogers, 2003). Our survey findings support this premise with 42% of students surveyed having ridden the bikes, compared to only 10% of faculty and staff. This finding is similar to previous cycling studies that found that younger adults are more likely to ride than older adults (Moudon et al., 2005; Shaheen et al., 2014). Specific to free-floating bike-share, the recent Seattle studies also found users to be younger (ages 18–44) (Hirsch et al., 2019a, Hirsch et al., 2019b; Mooney et al., 2019).

Another reason that freshmen and those living on campus were more likely to use the bikes may have been their physical location on campus. When the program was launched, the boundary (or geo-fence) for using the bike-share only extended to the perimeter of campus, and changed several times during the three-month period. Since point penalties were incurred for riding outside of the boundary, upperclassmen who lived off campus may have been less likely to use the bikes. The confusion about the rules of the program, as discussed during the focus groups, confirm this finding. People may have been willing to use the bike-share if they were given more information about where they could ride. Focus group student participants discussed how most users and non-users did not understand the “point system” incorporated into the program, and the geo-fence on the app was not readily apparent. This may have also explained the unintended consequence of bikes being parked incorrectly around campus and in the community. Social marketing approaches prior to launch may be needed to improve communication about new bike-share programs. Shaheen and colleagues in their 2010 review of bike-share highlight that successful bike-share systems communicate with users before system deployment (Shaheen et al., 2014)

According to survey data, staff and faculty had low uptake of the program as compared to students. During focus group discussions, staff and faculty expressed a lack of knowledge of the program, concerns about safety, and disapproval about the “littering” of bikes within the community. Hirsch and colleagues found similar barriers (Hirsch et al., 2019a, Hirsch et al., 2019b). To increase usage among faculty and staff, involving them in the planning prior to launch of a new program and understanding their concerns would be important. Staff and faculty may need more explanation of benefits the free-floating bike-share program offers. Communication about the convenience and health benefits of using the bikes and the resulting reduction in traffic, may be motivators for using the program.

Another study finding is that the relative advantage of using free-floating bike-share may have contributed to its uptake. Relative advantage is an attribute of the DOI theory that explains how improvements to what normally exists may increase innovation adoption (Rogers, 2003). In this case, walking, riding a personal bike, and using the university bus were methods of transport prior to bike-share. As discussed in several of the focus groups, the free-floating bike-share program provided a convenient one-way system of transportation that was more efficient than waiting for buses and less time-consuming than walking. The advantages of timesavings and convenience of the free-floating bike-share may have started a shift in transportation modes on campus. In a 2016 review of bike-share, convenience was a primary motivator of use (Fishman, 2016). Another study by Fishman and colleagues found station-based bike-share replacing sedentary activities but also replacing walking (Fishman et al., 2014). While a shift from walking to biking may mean less PA benefit for an individual trip, the study found an overall positive impact on PA due to bike-share (Fishman et al., 2014). The future of bike-share is uncertain as new innovations, like scooters and e-bikes, replace it (Shaheen and Cohen, 2019) and as limitations in infrastructure and funding exist (Hirsch et al., in press). Since the start of this study, the program has been replaced with free-floating bikes from another company. To date, other shared modes of travel, like scooters or e-bikes, are not permitted on campus.

Some in our study did not see the relative advantage of bike-share program. Because of the presence of a free bus system on campus, some students in the focus groups expressed that they preferred taking the buses to paying for free-floating bike-share. Staff and faculty, too, may not have felt there was an advantage to using bike-share over driving or walking. In a study of active commuting on a university campus, students were more likely to actively commute than staff and faculty, and staff and faculty did not feel that any intervention would encourage them to switch from driving to actively commuting (Shannon et al., 2006).

## 5 Strengths and limitations

Our study has several strengths. To our knowledge, this was the first study of free-floating bike-share on a college campus. The large number of bikes on a campus with a population of over 65,000 helps to provide a broad perspective on how free-floating bike-share launches may work at other universities. Further, the qualitative methods in this study reveal important contextual explanations for quantitative results. There were several limitations. The usage data provided by the bike-share company were restricted and limited our ability to see whether users were university-affiliated, single users, or super-users of the program (Winters et al., 2019). The survey was completed by <15% of the university community. There may have been systematic differences in those who responded versus those that did not. The current bike ridership question in the survey did not ask if current ridership was exclusive of bike-share riding, so it is unclear whether there was a mode shift from personal bikes to bike-share bikes. Studies have found, though, that those that currently own a bike are less likely to ride bike-share (Fishman, 2016). The focus groups were selected from this sample and may have differed from the larger university community. Since this was cross-sectional, we only examined initial uptake and not long-term adoption.

## 6 Conclusion

This study is a starting point in understanding issues around free-floating bike-share use in a university setting. For innovative bike-share programs to make a lasting health impact, future studies should address questions about the who, where, when, and why of bike-share use and the overall influence of bike-share on PA and health. Given that the average riding distance was only 0.5 miles, questions should explore number of rides per day and transportation mode shift over time. If a person uses bike-share regularly on a college campus will this translate into a habit of bike use and more PA in the future? Similar to other active transport modes, will bike-share riding be used as the “last mile” linking with other forms of transportation? Can and how would bike-share programs realistically replace car use? How will bike-share sustain with the introduction of other shared modes of travel, and how will campuses support multiple modes of travel? Bike-share interventions should address 1) marketing efforts to increase use and acceptance among later adopters and older populations, like faculty and staff; 2) education campaigns to increase understanding of rules of the programs and more acceptance from the broader community; and 3) infrastructure developments to improve safety and support wider use.

## Appendix A Supplementary data

Survey measures and focus group discussion guides Image 1
