# Supplementary material for: Evaluation of free-floating bike-share on a university campus using a multi-method approach
Source: Prev Med Rep. 2019 Aug 28;16:100981. doi: 10.1016/j.pmedr.2019.100981 (PMC6742965; doi:10.1016/j.pmedr.2019.100981)
Supplement: Supplementary file 1 — Survey measures and focus group discussion guides [file mmc1.docx]

| Survey Measures. | | |
| --- | --- | --- |
| Measure |  | Detail |
| Bike-share use |  | Staff, faculty, and students responded to the following questions: “Have you tried riding the [company name] bikes?” Possible responses were the following: no; yes, once; yes, 3-5 times; and yes, more than 5 times. For purposes of this analysis, yes responses were collapsed into a single yes category, so that the variable was a yes/no response dichotomous variable. |
| Campus residency |  | Students clicked on separate survey links depending on their residency status either off campus or on campus. |
| Employment status |  | Staff and faculty were asked if they were currently a staff or faculty member at the university. |
| Class rank |  | Student respondents were asked their class rank: indicating freshman, sophomore, junior, senior, or graduate student. |
| Age |  | Staff and faculty were asked to indicate their age. |
| Gender |  | Everyone was asked if they were male or female. |
| Current biking |  | Everyone was asked if they currently ride a bike and responded by choosing one of the following: yes, at least weekly; yes, but infrequently; no, but in the past year; no, but I did 1-5 years ago; No, it has been more than 5 years since I regularly rode a bike; no, I do not know how to ride a bike; and no, but there are times I wish I had acces to a bike. These responses were collapse into two categories: yes, currently riding and no, not riding. |
| Biking self-efficacy |  | Staff, faculty, and students were all asked how confident they were that they could ride a bike for one mile safely on campus. Responses included the following: very confident, confident, neither confident or unconfident, not confident, and not very confident. The very confident and confident responses were collapsed into a yes confident response, and the neither confident or unconfident, not confident, and not very confident responses were collapsed into a not confident response. |

| Focus Group Discussion Guides. | |
| --- | --- |
| Focus Group Type | Discussion Items |
| Participants who had tried the bike-share on campus | Why did you first try riding the bikes? |
|  | How often do you use the bike-share bike and for what purpose? |
|  | What other mobility modes did you use to get around campus before the bike-share program? |
|  | What do you like better about the bike-share than other mobility modes around campus? |
|  | What do you like less about this bike-share than other mobility modes around campus? |
|  | How could the bike-share be improved? |
|  | Explain what you think to be the “rules” of the bikes. What are your thoughts about the “rules” (racking the bike, going off campus, etc.)? What would help in getting users to be more compliant with the “rules”? |
|  | Describe your thoughts about safety with the bikes. |
| Participants who had **not** tried the bike-share on campus | How do you usually get around campus? |
|  | Have you seen the bike-share bikes on campus? |
|  | Did you know that this university has a bike-sharing program on campus? Talk about what you know about the program (cost, sign-up, drop-off, area). |
|  | What would encourage you to try bike-share? |
|  | Why would you not try bike-share? |
|  | Explain what you think to be the “rules” of the bikes. What are your thoughts about the “rules” (racking the bike, going off campus, etc.)? What would help in getting users to be more compliant with the “rules”? |
|  | Describe your thoughts about safety with the bikes. |
